# The Impact of the Fourth Industrial Revolution on Managers’ Sense of Coherence

**DOI:** 10.3390/ijerph18083857

**Published:** 2021-04-07

**Authors:** Claude-Hélène Mayer, Cemonn Wegerle, Rudolf M. Oosthuizen

**Affiliations:** 1 Department of Industrial Psychology and People Management, College for Business and Economics, University of Johannesburg, Johannesburg 2006, South Africa; cemonnwegerle@gmail.com; 2 Kulturwissenschaftliche Fakultät, Europa Universität Viadrina, 15234 Frankfurt, Germany; 3 Department of Industrial and Organisational Psychology, School of Management Sciences, College of Economic and Management Sciences, University of South Africa, Pretoria 0003, South Africa; oosthrm@unisa.ac.za

**Keywords:** Fourth Industrial Revolution (4IR), salutogenesis, sense of coherence, mental health, managers

## Abstract

The Fourth Industrial Revolution (4IR) disrupts the world of work, new technologies change the nature of individuals’ work and their tasks, and therefore it is necessary to determine how managers cope with these changes, specifically relating to their salutogenesis. There is a lack of research conducted on the salutogenesis of managers in times of the 4IR. The purpose of this study is to investigate the level of managers’ sense of coherence (SOC) in terms of the adjustments and developments of the 4IR, and their in-depth understanding of their SOC. This study employs a hermeneutical research design with a qualitative approach by using a semi-structured interview. The method used to analyze the data was content analysis. From the data analysis, the findings indicate that a majority of the managers tend to have an understanding of the 4IR and what implications of the 4IR will have on the world of work and their job description, the necessary resources to cope with the 4IR, and find the 4IR meaningful, therefore, managers have a strong SOC level during the 4IR. The recommendations for future studies suggest that research could be conducted how managers and lower-level managers’ SOC differ, which will provide insight into what different methods are required for the different level of managers.

## 1. Introduction

Technological advances are witnessed through history, where humans implement technology to address the limitations of human practices [1]. Skilton and Hovsepian [1], state that technology is utilized to build machines that aid in the achievement of specific tasks or substitute human work. Consequently, industrial revolutions emerged to reshape the production of goods and services through innovative technology. In addition, the Fourth Industrial Revolution (4IR) is where technology reflects rapid innovation and technology transformation [2]. This revolution has a great impact not only on the work processes but on the individual as well. Hattingh [3] (p. 8) highlights that due to the rapid velocity change and new disruptive technology of the 4IR, the working environment is “unknown and unpredictable”. Hattingh [3] further mention that the changes of the 4IR have led to substantial job loss of unskilled employees and personal trauma as workers battle to cope with vulnerability and precariousness of the new world of work.

In addition, Coldwell [4] mentions that the 4IR has a negative effect on the mental health of managers. However, there is not adequate literature relating the effect of 4IR on the mental health of the workforce. The question arises of ‘is the workforce confident and prepared to deal with the changes brought by the 4IR?’ Therefore, research on the mental health of managers, specifically focusing on salutogenesis, is necessary to fill the gap. Antonovsky’s [5] theory of salutogenesis focuses on the sense of coherence (SOC) of individuals. SOC is defined as: “a global orientation that expresses the extent to which one has a pervasive, enduring though dynamic feeling of confidence that one’s environment is predictable and that things will work out as well as can reasonably be expected” [6] (p. 11). It is necessary to measure the manager’s SOC in times of the 4IR to better provide assistance through the change. Therefore, by measuring the managers’ SOC will determine their mental health, which indicates the preparedness of managers to deal with change.

### 1.1. Mental Health in the Fourth Industrial Revolution

Throughout history, industrial revolutions have brought changes in the economy, society and the world of work, where the industrial revolutions have had a dire impact on all aspects of society, work and life in general [7]. Industrial revolution is a term that refers to an economic change that is characterized by a new era of capitalist development or a point of departure to ensure economic growth and maturity [8]. The 4IR is the integration of evolving technology into the physical and biological worlds. This has not been the case in the other industrial revolutions with advances increasing at an exponential rate [1]. Dombrowski and Wagner [9] comment that the new key technologies will inevitably result in job losses, redundancies and de-industrialization. Webber-Youngman [10] lists the skills needed to adapt to the 4IR as critical thinking, problem-solving, creativity and innovation, emotional intelligence, cognitive flexibility and adaptability. This implies that individuals need to adapt their skills and knowledge to thrive in the new world of work in the 4IR. According to [4], the strain experienced to retain employment in the changing and dynamic 4IR environment has an adverse effect on the mental health of managers. These changes influence individuals’ employability and their working environment, which in turn influence their mental health [4].

Various theories exist that try to explain the relationship between the mental health of managers and their working environment [11]. Unfortunately, most theories focus on the negative effects on mental health, whereas Antonovsky’s [5] theory tries to determine what creates positive mental health, rather than is harmful to mental health in situations of suffering and stress, this is known as salutogenesis [12]. The focus is on the individuals’ feeling of assurance that their internal and external environments are assured and events will proceed as planned [13]. In Germany, Japan and the US, the 4IR is perceived as an opportunity rather, than as a threat [14], whereas in South Africa (SA) it is generally seen as a threat since the challenges of the 4IR are likely to cause job losses. In developing countries such as South Africa, this can be serious because of the high unemployment rates [15]. In some countries the 4IR has been known to cause hardship, therefore, an investigation into how managers stay healthy during the changes brought about by the 4IR. It follows that a positive attitude is required to ensure that managers will strive to understand, manage and fulfill the opportunities and challenges of the 4IR meaningfully.

The 4IR has also prompted disruption in society as witnessed in the preceding industrial revolutions. As technology develops exponentially, the repercussions on the workforce are noticeable. The 4IR builds on the technological advances of the Third Industrial Revolution (3IR), which integrates the physical, digital and biological worlds [16]. The by-product of the innovative technology used in the 4IR offers numerous opportunities and challenges. This industrial revolution is set apart from the previous industrial revolutions by the dissemination, speed and the scale of the new technologies which have been implemented in various sectors. Li et al. [17] classified the technologies as (1) digital technologies include the internet of things (IoT), artificial intelligence (AI), machine learning, big data and cloud computing, as well as digital platform, (2) physical technologies include self-driven cars and 3D printing, and (3) biological technologies include genetic engineering and neurotechnology. 

Each industrial revolution brought certain changes to the world of work that affected managers in various ways. Furthermore, although the previous industrial revolutions saw the creation of new and the redundancy of jobs present managers adapted to this change. However, the 4IR is different from the previous industrial revolutions in terms of the exponential evolving pace and the depth to which changes are occurring in the world of work [18]. Prisecaru [19] discusses the impact of the 4IR on the economy and society, where self-employment, short-term contracts and part-time workers are key players in the disruption brought by this industrial revolution. Prisecaru [19] further states that the disruption of the 4IR in the labor market not only affects the individuals, but tax revenue, pension funds and the gross domestic product (GDP). In addition, Prisecaru [19] contents that the 4IR will affect the income distribution with the low-income earners being seriously affected, and pensions being lower. Li et al. [17] on the other hand, argue that the 4IR creates greater opportunities for future economic development, with an increase in production efficiency and a shift in the global value chain.

One of the strengths of SA is innovation owing to an active innovative culture and entrepreneurial activity. However, Levin [20] highlights concern regarding preparation of South Africa’s human capital for the 4IR. In addition, Levin [20] asserts that it is essential that the institutional framework of SA be reviewed and improved to facilitate the change to 4IR thereby ensuring an environment with steady policies that can direct innovation.

### 1.2. The Effect of the Fourth Industrial Revolution on the Organisation and Workforce

With the new technologies proliferating in the economy, society and industry, these technologies have had major consequences in everyday life and the world of work. Brondoni and Zaninotto [21] opine that the 4IR has forced a shift in businesses that has altered the traditional business and organizational models. In turn, the labor market will be affected by this industrial revolution that offers more flexibility and on-demand work [3]. Furthermore, Hirschi [22] cites the loss of jobs, the change of occupation and the emergence of new occupations, resulting in the relocation of power, wealth and knowledge [16].

The question is how organizations will support managers’ mental health during the time of the 4IR? Organizations generally implement programs to promote mental health and address stressors in organizations, namely stress management interventions and/or workplace health promotion strategies [23]. Salutogenesis is the application of resources and outcomes that relate to the positive health-orientated change process in the organization [23]. However, there is a lack of information on how organizations can assist their managers during the 4IR to promote salutogenesis. A study conducted by Morathi [24] in an information technology company, the managers formulated the following guidelines to assist them during the changes brought about by the 4IR, which will enable them to be more self-sufficient, to develop the managers, provide future-orientated leadership, to practice transparency, and to give clear descriptions of their future roles.

Min et al. [25] determined the occupational health issues relating to the 4IR. With automation and robots taking over repetitive or simple tasks, managers experience job insecurity, and this instability provoked by 4IR can give rise the mental illnesses among managers [25]. Although the 4IR technologies put forward an argument for increased productivity and a better quality of life, Min et al. [25] argue that automation results in increased human labor time to improve productivity and keep abreast with the competitive firms, thus resulting in increased occupational stress. When Dombrowski and Wagner [9] looked at the mental strain of socio-technical production systems on imposed managers, they observed that sophisticated problem solving was necessary and managers would require different competencies to function in the 4IR. From the secondary analysis used to gather the information, it is evident that work systems that are subjected to change (such as 4IR) give rise in an increase in the mental demands process related to working systems [9]. 

Furthermore, in South Africa, the 4IR has brought in new employment equity (EE) practices that will govern the effects of this revolution [26]. Oosthuizen and Mayer [27] (p. 2) state that as “job mobility, consent retraining and rotation” are necessary to improve managers’ flexibility, employability and suitability in today’s world of work, an adaptation to EE is necessary to address the changing work environment. According to Min et al. [25], government policies should also be adapted to protect the health rights of managers who are faced with increased atypical employment due to the 4IR, currently some managers are not protected by the labor laws [25]. Finally, the 4IR can increase the quality of managers’ lives or increase the risk of unemployment [28]. However, in both cases, it is imperative to remain mentally healthy, although these situations can result in improving or loss of quality of life. 

### 1.3. Salutogenesis and Sense of Coherence

Traditionally health theories focused on pathogenesis, which looks at the origin of the disease to avoid or limit the spread of disease [29]. When Antonovsky [5,30] sought the answer to the question of what keeps people healthy, he introduced the concept of salutogenesis [13]. Schnyder et al. [31] state that salutogenesis focuses on maintaining good health amid undesirable stressors. Salutogenesis focuses on the origin and assets of health, and the central focus of the Salutogenesis Model is SOC, which is molded by an individual’s life experiences [32], therefore, it focuses on the resources and factors that will generate good health and wellbeing [33]. The resources used to develop one’s SOC are referred to as general resistance resources (GRR), which are measures that one implements or uses to relieve stress in challenging times [33]. GRR include social support or support networks, knowledge, self-esteem and money [34]. 

Antonovsky [5] (p. 168) defines SOC strength “as a global orientation that expresses the extent to which one has a (A) pervasive, enduring, though dynamic feeling of confidence and that stimuli, deriving from one’s internal and external environments in the course of living are structured, predictable and explicable; (B) the resources are available to one to offset the demands posed by these stimuli; and (C) these demands are challenges worthy of investment and engagement”. Therefore, a strong SOC assists individuals to marshal resources to manage stressors and change effectively [32]. SOC includes the following dimensions: comprehensibility (cognitive element), manageability (behavioral element), and meaningfulness (motivational element) [35,36,37]. Comprehensibility is a concept that refers to the rationalization of external and internal elements to make sense, which results in the predictability of the elements or the possibility of implementing problem-solving strategies [38]. Manageability relates to the individuals’ believes or confidence in their personal and external resources ate their disposal are ample to address the demands of internal or external stressors experienced [38]. Meaningfulness is the perception of whether the challenges encountered are deemed as a worthwhile endeavor [38]. Therefore, individuals with high SOC levels: “wish to, be motivated to, cope; believe that challenge is understood; believe that resources to cope are available” [6] (p. 15). 

Braun-Lewensohn and Mayer [39] elaborate that SOC is an appraisal of the environment to assisting the examination of the available resources that can be utilized to cope with a stressful event or situation. GRR also includes coping strategies, which are defined as behavioral factors that summon the effort to be implemented to make a stressful situation or stressors tolerable and to minimize the negative effects of the situation [39]. SOC is considered as being flexible, therefore, “not constructed around a fixed set of mastering strategies, like the classic coping strategies” [36] (p. 241). Furthermore, the SOC is a tool to measure individual’s ability of resilience when facing challenges or stressors. In other words, the SOC measures the ability to implement the appropriate coping strategies and processes. Therefore, individuals with high scores of SOC will effectively implement strategies to address encountered stressors, while individuals with low SOC scores display the opposite [31].

### 1.4. Salutogenesis in the Fourth Industrial Workplace

The SOC measure is also applicable to both the individual’s working life. Bauer and Jenny [40] define a healthy organization as one which is characterized by producing both low pathogenic processes and high salutogenic processes. The focus of organizational health development (OHD), should be where health in organizations is improved and maintained through the interaction between the individual and the organization’s capacities including salutogenic health development. This is achieved by explaining critically the managers’ working environment and their SOC. The framework of salutogenesis is based on two concepts, which are the SOC and GRR, and the specific GRR in the work context includes job control, task significance and social relations [37].

As it is evident that the 4IR is accompanied by dynamic changes which affect both the organization and the individual, thus attention should be paid to the health promotion in the organization to bring about change. Change involves the complex relationship between psychosocial elements (objective procedures and subjective experiences), job design, policies and procedures and the external environment of the organization [41]. The model of salutogenesis can be used as an intervention to foster organizational health development [40]. Furthermore, these interventions can be used to address job demands (work-related stressors) and job resources (work-related GRR) to ensure a better balance between stressors and GRR to improve the work experience of managers [40]. 

Studies have been conducted on determining the effect of SOC on burnout, work engagement, personal accomplishments, and occupation stress [35,42]. Another study conducted by Mayer [13] aims to assist in the development of South African managers’ SOC. In addition, a study conducted in the Democratic Republic of Congo, investigates the SOC, burnout and coping, which contribute towards positive psychology in African literature [43]. Mitonga-Monga and Mayer [44] reported that individuals were dedicated and engaged when they observed their world of work as structured. Recent studies have focused on the connection between the work-SOC and the SOC’s prediction on work engagement, stress and manager wellness [43], and the link between the SOC, mindfulness and the Big Five personality traits [45]. The study conducted by Van der Westhuizen [43] found that work-SOC significantly predicted work engagement and fatigue. The study conducted by Grevenstein et al. [45] concluded that SOC and mindfulness showed incremental validity in the Big Five traits, therefore, a significant correlation existed. Pallant and Lae [46] assert that individuals with higher levels of SOC are more inclined to cope with stressors by using adaptive strategies.

Various literature has examined the effects of the 4IR on individuals and to gather a greater understanding of the 4IR [4,22]. However, a lack of research is conducted on managerial managers’ sense of coherence levels during the 4IR [26]. Although a study [26] was conducted to determine how salutogenesis can assist international leaders to cultivate healthy organizations, research lacks to address the question of how various levels of South African managerial managers are functioning during the 4IR. The 4IR creates various technological changes that will disrupt how a business operates and generate value, the way people work and live [3,47]. As a result of this industrial revolution, the nature of individuals’ work and their tasks will change [22], therefore, it is necessary to determine how managers cope with these changes, specifically relating to their salutogenesis. Furthermore, to manage change and challenges one needs to have a strong salutogenesis to comprehend, manage it in a resourceful manner, and see the meaningfulness in that change and new job requirements. 

The purpose of this study was to investigate the level of managers’ sense of coherence (SOC) in terms of the adjustments and developments of the 4IR, and to gain an in-depth understanding of the three SOC components, namely comprehensibility, manageability and meaningfulness. The study also determined how managers with high and low SOC level scores manage the stressors inherent in the 4IR. 

## 2. Materials and Methods

The nature of inquiry of this study employs an interpretivist approach, which is implemented in this study to provide a detailed understanding of the salutogenesis of managerial managers in South Africa and their interpretation of the era of the 4IR.

### 2.1. Research Design and Approach

The research design applied in this study was a hermeneutical design, which refers to the understanding and interpretation of texts such as organizational activities or events, the goal is to derive the participants’ meaning from the text [48]. As a qualitative interview method was employed to gather primary data in the qualitative research paradigm, a hermeneutical approach was adopted.

### 2.2. Sample

As the population under investigation consists managerial employees in South Africa, hence the sample consisted of altogether 17 managers from various industries. Out of the 17 managers, 10 were male and 7 were female, ranging in age from 26 to 63 years. For 10 individuals, Afrikaans was the first and English the second language while for 7 individuals English was the first language. The industries managers worked in range from manufacturing, human resource management, information technology finance, retail, construction, professional services to health and care services. Positions in the organizations were: Executive Director, Senior Executive, Senior Research Officer, Financial Director, HR Managers, Head Accountant, Commercial Director, and Medical Sales Manager.

The sampling methods used were purposive and snowball sampling [49,50]. Purposefully chosen, specific individuals were invited to participate in the study. These individuals were directly contacted by email or telephone. 

Data were collected from selected managers on lower, medium and upper managerial levels in different organizations. All of the organizations, managers were recruited from for interviews are organizations which are transforming into 4IR workplaces. Selection criteria were therefore: (1) managers on different managerial levels; (2) fluently in English language; and (3) working in organizations which are in the process of transforming or are transformed in terms of the 4IR. 

### 2.3. Data Collection and Data Analysis

One of the researchers conducted semi-structured interviews with the managers. Questions in the interviews included, for example: “how do you perceive the 4IR process?”; “tell me about detailed experiences of the 4IR in your organization.”; “what are your personal resources to cope with the 4IR?”; “how do you manage the 4IR processes?” and “what makes your life meaningful in the 4IR context?”.

The method of analysis was the interpretive qualitative content analysis [51]. The interview texts were coded and categorized [52] by an abductive approach where the themes were established prior to the data analysis through categories formed from the analysis of the data [53].

### 2.4. Quality Criteria

The rigor of qualitative inquiry [54] is necessary to establish that the results are evidence and scientific based [55]. The trustworthiness of the research was ensured by adhering to the requirements of credibility, transferability, dependability, and conformability [55,56,57,58]. Credibility was addressed by presenting research and findings in transparent ways which fulfil the objective of the study and focusing on selected characteristics [58], through reading and re-reading the data to identify codes and thereafter formulated themes. Dependability was determined by the reliability of the findings or the consistency of the results, which is guaranteed by maintaining records (audit trail) of the steps followed and conclusions made during the research process [54,58]. Transferability was achieved by that the findings can be repeated in the same context with the same participants (no ambiguity found in decisions made) [56], by using a thick description of the research process [54,58]. Conformity was achieved by that the results were verified and supported by other researchers [59], by describing the process in detail and documenting the records [54,56,58].

### 2.5. Ethical Considerations

In terms of ethical considerations [60], the participants were provided with the information relating to the study and given a choice to participate in the study [61]. The participants were informed that they had the right to withdraw from the research process at any stage. While the participants were provided with the necessary information regarding the objectives of the study [62], the participants were provided with the opportunity to exercise their right of informed consent. Confidentiality was obtained by guaranteeing participants’ anonymity and not disclosing any identifiable information from the participants [63]. Precautions were put in place to prohibit the disclosure of personal information to irrelevant parties. While reporting was conducted in a transparent and honest manner to avoid deception [61], the researchers did not draw conclusions that were not supported by the data gathered [64]. 

## 3. Results

The results of the semi-structured interviews on the SOC during the 4IR is provided with direct quotations.

### 3.1. Theme: Comprehensibility

Managers were asked to describe the 4IR and most managers understood the 4IR as technological development by naming the disruptive technologies (see Table 1). Where 12 out of 17 participants named examples of these new technologies, and 10 out of 17 participants mentioned that the 4IR is disruptive technologies:

*“Uhm, and actually quite disruptive, I think we hear about stuff like water, you know, self-driving cars, and maybe household appliances being interconnected, everything…” (Participant 2, Male). Hence the participant is describing the 4IR by proving examples of the new technologies of the 4IR namely self-driving cars.*

Furthermore, the participants were asked to comment on how their job descriptions will change due to the 4IR and 13 out of 17 participants mentioned that their job descriptions will remain the same, however, additional practices will be required:

“*So, I think that would probably say the same. Uhm, the only thing that would they’ll probably add some auxiliary in front of it, like online research, or virtual research or something like that…*” (Participant 3, Male). In other words, the participant believes his job description would not be entirely affected by the 4IR, but rather additional skills will be needed. 

Furthermore, participants understood that the developments of the 4IR will mostly impact their life and health in a positive way:

“*It’s made my life easier. I get here, I didn’t have I didn’t have[sic] a computer when I started here, or my other companies where I worked, I had a computer to keep in touch with uhm, people overseas or contacts…*” (Participant 14, Male). This participant articulates the comfort and improvement of life due to the new technologies. 

“*Now it is quite nice, because you book your class, and then you know, you’re in that class, you can plan your day around that class. So, I think it’s helped me… uhm dedicate better to do physical activity.*” (Participant 9, Female). Therefore, the participant pronounces the easiness of exercise due to the new technological developments.

### 3.2. Theme: Manageability

This theme outlines the resources used to cope and manage the changes of the 4IR (see Table 2). Most participants mentioned that skills and knowledge are important resources to have to remain relevant during the 4IR. Therefore, continuous learning, connecting with others, upskilling, adaptability and observations are important practices during the 4IR:

“*Yes, I would say I read a lot, I read a lot. Uhm, I read, uhm, a lot of articles related to my job, and also to the technology.*” (Participant 7, Female). This illustrate that continuous learning is necessary to remain relevant during 4IR. 

“*And other thing as well is connecting with people face to face, virtually, and learning from their experiences and then determining, you know, what is, you know, how can I respond to that, to ensure that they have uhm, a good experience.*” (Participant 6, Female). Hence, tapping into other professionals’ knowledge and skills to manage the changes of the 4IR, which emphasis interpersonal skills. 

“*Uhm so ja, I think just basically people that are stuck in their ways are not going to cope [laugh] they will have to be adaptable.*” (Participant 2, Male). This statement demonstrate that adaptability is needed during the 4IR, otherwise people will not cope with these new changes.

### 3.3. Theme: Meaningfulness 

The participants view of the 4IR is overall positive and optimistic about the changes brought by the 4IR (see Table 3):

*“I think it’s a positive aspect… I think it’s like some strain off human on humans.”* (Participant 6, Female), in other words, the 4IR improves the efficiency of human tasks. 

*“Oh, yes, for sure. I think it’s probably the best thing. Yeah, the best thing we can do now is to push forward with it.”* (Participant 13, Male). Therefore, the 4IR is viewed in a positive light and it is to the benefit of people to develop with the 4IR. 

Furthermore, participants were asked to describe will make their job meaningful, and 13 out of 17 participants mentioned that technology can assist in making their job meaningful:

“*…I can reach more people through technology that would be great, uhm that I could get the knowledge out there. Uhm, you know, get more knowledge to more people*.” (Participant 5, Female). This indicates that employing technology to be of service to people is an important aspect in the individual’s job. 

## 4. Discussion

The general aim of this study was to determine how do managers in South Africa conceptualize the 4IR as a result of their SOC levels. The overall results of comprehensibility illustrate that majority of the managers tend to understand the 4IR and changes brought about in their job descriptions. The results illustrate that the most frequent understanding of the 4IR is the technological component, namely the development of disruptive technologies. These descriptions are consisted with international literature of Schwab [65], who describes the 4IR as a technological revolution which brings about changes and transformation. Furthermore, digitization and access to information are also used to describe the 4IR, which is similar to that of Griffiths and Ooi [2], Hirschi [22], Lasi et al. [66], Lombard [67], Skilton and Hovsepian [1], and Schwab [65], which include unlimited access to information, digitization and the fusion of biological and physical worlds. 

However, the results also indicate that the human element cannot be fully removed from the workplace, with Paba and Solinas [68] sharing the opinion of the participants that not all human tasks can be replaced by the new technology, therefore, these results contribute to international literature. The results also indicate that majority managers mostly believe that their job descriptions will not change due to the 4IR in the near future, but additional skills will be needed to stay relevant during the 4IR. These results are similar to the South African findings of Abdulla [69], where managerial activities and leading of people is secure from job automation. 

Furthermore, the results demonstrate that the managers believe they have the necessary resources to manage the challenges and change brought by the 4IR, where the manager’s emphasized the resources of having the relevant knowledge and skills to remain relevant during the 4IR. The international results of Güleryüz and Duygulu [70] also demonstrate that the leaders prepare themselves for the 4IR by gaining the necessary knowledge and skills, and understanding of the 4IR. 

In addition, the results of meaningfulness indicated that managers find that the 4IR meaningful due to the majority of the changes seen as a positive and the optimistic. Similar to Abdulla (2019), who found that most South African managers had a positive view of the technologies available due to the 4IR, however, negative views were also shared. In addition, Mayer and Oosthuizen [26] found that international managers tend to focus on positive elements when working with challenges during the transition into the 4IR due to their substantial focus on a strong Sense of Coherence, salutogenesis and meaningfulness. Furthermore, majority of managers’ work is meaningful when they are able to utilize technology to reach more people and upskill them. It is evident that the 4IR can assist managers in making their job meaningful. Findings of Mayer and Oosthuizen [26] suggest that international leaders find meaningfulness important, which result in the necessary strengths and motivation during 4IR, however, literature on what makes manager’s work meaningful is scarce. 

The findings do not necessarily provide a critical view on the 4IR, but rather reflect the experiences and insights of the managers with regard to the changing environments within the framework of SOC.

The limitations of this study entail the subjective perspective on the qualitative approach that results in the probability of the researcher’s bias to reflect certain data. As usual in qualitative research, the findings are not generalizable. The study is limited to a relatively small sample size of 17 individuals. Another limitation of the study is that the sample is not representative of all South African cultures, since the sample consists mostly of white participants, with the majority of the participants being male (10), whereas the female participants number seven of the seventeen participants. Furthermore, these findings cannot be linked to a specific industry since participants work across industries. 

## 5. Conclusions and Recommendations

The 4IR incites disruption in society, the economy and industry by introducing dynamic changes, which will affect businesses, individuals and their jobs. To cope with these changes and challenges one needs to have a strong salutogenesis to comprehend and manage change in a resourceful manner and to see the meaningfulness in that change and the new experiences at work. Considering how extensively the 4IR is discussed and the effects of the 4IR on economies, businesses and jobs, there is there is a noticeable lack of research on the Salutogenesis of managers at the time of the 4IR. Consequently, this research study was employed to explore managers’ SOC levels with relevance to the 4IR. 

It can be concluded that managers try their best to understand the 4IR well, to grasp the concepts and to see its application in their daily managerial work and activities. Further, they believe that they need resources to manage rapid changes and stay at the top of the trend. Finally, managers use a positive psychology perspective: they aim at seeing the meaningfulness in the new trends of the 4IR. That does not mean that they are uncritical, but it rather shows that they try to adapt to a change that is unstoppable. While they are trying to make ends meet, managers ascribe meaningfulness into the new work life which is strongly influenced by technology. They define certain 4IR trends therefore as a meaningful chance to reach a growing number of people (clients, stakeholder, colleagues, competitors etc.) and to be stimulated through the experience of new effects of the environment on the organization and the self. They further see meaningfulness in their own upskilling and their own familiarization with the new challenges and the response to the question of how to handle the new trends.

Managers tend to have a good understanding of the 4IR by describing this concept according to other literature. In addition, managers predict that their job descriptions would not change in the near future, but additional practices will be required. Managers consider they have the necessary resources to manage and cope with the changes and challenges that emanate from the 4IR. Furthermore, managers find their job meaningful when technology can be used to help and upskill others. Furthermore, according to the findings, managers perceive the 4IR as an overall welcome change. A majority of the research focuses on how the 4IR impacts occupations of specific nature, and the effect on organizational structure, economies and societies. However, literature on positive mental health remain scarce in the 4IR field. The recommendations for future studies suggest that research studies could be conducted how managers and lower-level managers’ SOC differ, which will provide insight into what different preparation methods are required for the different level of managers. Furthermore, these findings could be used as a basis for future studies, which employs a quantitative approach to generalize the findings to a bigger population. On a practical level, organizations should focus on building comprehensibility, manageability and meaningfulness by addressing their human resource management, adjusting their training programs and supporting their managers by building a meaningful and manageable work culture based on cooperation, openness and trust.

## Figures and Tables

**Table 1:** The Comprehensibility of the Fourth Industrial Revolution (4IR) by Managers.

Frequency	Code	Participant	Category	Frequency
12	Naming 4IR technologies	P1, P2, P3, P4, P5, P6, P8, P9, P10, P11, P12, P14	Technology	22
10	Disruptive technology, development of technology	P2, P3, P4, P5, P8, P9, P10, P14, P15, P17		
13	Job descriptions stay the same—additional practices.	P2, P3, P4, P5, P6, P7, P9, P10, P11, P12, P14, P17	Additional practices	26
8	Life will be easier and more comfortable	P4, P7, P8, P10 P11, P13, P14, P15	Positive impact	29
8	Good impact on health	P1, P6, P9, P10, P11, P12, P13, P17		
7	Technology improved monitoring of exercise.	P1, P6, P8, P9, P11, P12, P14		
6	Good impact on health sector	P7, P8, P10, P11, P12, P17		

**Table 2:** Manageability Resources Used by Managers.

Frequency	Code	Participant	Category	Frequency
12	Skills and knowledge	P1, P2, P3, P5, P7, P10, P11, P12, P13, P14, P16, P17	Skills and knowledge	62
12	Others’ skills and knowledge	P1, P2, P3, P4, P5, P6, P7, P9, P12, P13, P14, P16, P17		
12	Continuous learning	P1, P3, P7, P8, P9, P10, P11, P12, P13, P14, P15, P17		
9	Upskilling	P4, P5, P6, P7, P10, P11, P12, P15, P17,		
9	Stay informed	P1, P3, P4, P6, P7, P8, P9, P15, P17		
6	Adaptability	P2, P3, P6, P9, P14, P15		
2	Observation	P2, P16		

**Table 3:** Meaningfulness of the 4IR Perceived by Managers.

Frequency	Code	Participant	Category	Frequency
17	4IR is positive	P1, P2, P3, P4, P5, P6, P7, P8, P9, P10, P11, P12, P13, P14, P15, P16, P17	Positive view	50
14	4IR positive and negative	P1, P2, P3, P4, P5, P7, P8, P11, P12, P13, P14, P16, P17		
14	Optimistic about 4IR	P1, P2, P4, P6, P7, P8, P9, P10, P11, P12, P13, P14, P15, P17		
8	Reach more people through technology—provide more knowledge and skills	P2, P3, P5, P8, P10, P12, P13, P14, P15	Technology	14
2	Providing more access to psychological services	P3, P12		
4	4IR tool in Meaningfulness	P1, P4, P6, P9		

## Data Availability

The data presented in this study are available if requested.
